# Bridging policy and practice: Adapted physical education for special needs learners in BRICS

**DOI:** 10.4102/ajod.v14i0.1626

**Published:** 2025-09-04

**Authors:** Charlene Engelbrecht, Dorita du Toit, Nico van der Merwe, Chanelle Kemp

**Affiliations:** 1 School of Psychosocial Education, Faculty of Education, North-West University, Potchefstroom, South Africa

**Keywords:** adapted physical education, BRICS countries, special educational needs, inclusive education, disabilities

## Abstract

**Background:**

Adapted Physical Education (APE) focuses on tailoring school Physical Education (PE) to the needs of learners with special educational needs (LSEN), promoting their optimal physical and psychosocial development, which is also one of the priorities of the Brazil, Russia, India, China and South Africa (BRICS) organisation in addressing the health issues of its people. In view of limited available literature, more research is needed on the state and status of APE in BRICS.

**Objective:**

To explore the current state and status of APE in the BRICS countries.

**Method:**

Employing the qualitative document analysis methodology, school curriculum documents and education policies in the BRICS countries were analysed inductively within an interpretivist perspective, based on the frameworks of Bronfenbrenner’s Ecological Systems Theory and the Cultural-Historical Activity Theory.

**Results:**

Although BRICS policies mandate the inclusion of LSEN in PE in mainstream and special schools, specific guidelines for adapting activities in PE could only be found in governmental documents of Russia, India and China. Teachers of LSEN are required to be qualified in special or inclusive education, but specialised undergraduate and professional APE teacher training is not equally available in all BRICS countries. Specific requirements regarding the assessment of LSEN within APE are also lacking.

**Conclusion:**

More comprehensive guidelines are needed regarding teaching strategies, curriculum content, assessment and professional development in APE to address challenges in APE implementation across BRICS countries.

**Contribution:**

Implementing the recommendations of this study would enhance the physical and psychosocial development of LSEN in BRICS countries.

## Introduction

Health and education are two of the investment priorities of the original BRICS, a collaborative organisation consisting of Brazil, Russia, India, China and South Africa (Egypt, Ethiopia, Iran and the United Arab Emirates joined the organisation in January 2024, and now it is called ‘BRICS +’) (BBC 2024; O’Neill 2021). The main objectives of the BRICS organisation include economic cooperation, economic growth, global influence and human development among its member nations, of which citizens’ well-being in terms of their education and health are essential components (BBC 2024; O’Neill 2021; Van Jaarsveld 2021). In light of BRICS’s health development goals, the global increase in disabilities, shown in the 2017 Global Burden of Disease Study, which included a 17% increase in physical rehabilitation needs (the optimisation of physical function and participation in persons with physical impairments such as cardiovascular disease and autism) in the BRICS countries (Jesus et al. 2020), is concerning. One way of rehabilitating and addressing physical disabilities and other special education needs such as intellectual disabilities is by means of Adapted Physical Education (APE), which is essentially inclusive or specialised Physical Education (PE) (Block et al. 2021). Adapted Physical Education creates an avenue through which learners with special educational needs (LSEN) can be offered equal opportunities in schools to actively participate and develop physically (Barros, Coelho & Martins 2023; Gonçalves, Leite & Duarte 2020).

Besides physical and motor fitness benefits, LSEN also benefit from APE through having an improvement in their psychosocial health (Dheesha 2017). However, research in the field of APE in the BRICS countries show that LSEN often do not participate efficiently during PE classes and do not experience social inclusion, because of several challenges in policy implementation (Costin & Pontual 2020; Gale et al. 2022). In this regard, studies in individual BRICS countries show that challenges faced in the implementation of APE include: PE teachers who are not trained to work with LSEN, LSEN not always having access to appropriate learning environments because of the lack of needed accommodation and LSEN often feeling socially isolated (Biktagirova & Korotkova 2016; Block et al. 2021; Burnett 2021; Chennapragada 2021; Gonçalves et al. 2020). However, no study could be found comparing APE across all five nations, indicating a gap in the literature that this study aimed to address.

The ecological systems of a country, including mixed governance systems and national policies on inclusive education and school curricula, play a major role in these challenges experienced in APE in schools. Furthermore, as the activities of LSEN are dependent on certain social and cultural contexts and can be assisted by specific tools within these ecological systems, the study was conducted within the theoretical frameworks of the Bronfenbrenner’s Ecological Systems Theory (EST) (Bronfenbrenner & Ceci 1994; Bronfenbrenner & Morris 2007) and the Cultural-Historical Activity Theory (CHAT), which will be discussed next.

### Theoretical frameworks

Bronfenbrenner’s EST suggests that an individual’s learning and development are influenced by the regular and increasingly complex interactions between the learner and his or her immediate environment (Bronfenbrenner & Ceci 1994; Bronfenbrenner & Morris 2007). According to Bronfenbrenner and Morris (2007), the immediate ecological environments are conceived as a set of layers, which consists of the microsystem, the macrosystem, the mesosystem, the exosystem and the chronosystem. The microsystem involves the immediate environment of LSEN, which includes his physical, social and interpersonal relationships (Bronfenbrenner & Morris 2007). The microsystem also refers to the child’s disability, child’s attitude towards his or her ability as well as the child’s willingness to be supported (Bronfenbrenner & Morris 2007; Meltzer & Muir 2022). The mesosystem refers to the relationships that exist between the different microsystems (Bronfenbrenner & Morris 2007). The mesosystem thus includes support to LSEN and the learning and development of LSEN (including direct contact with other learners in the classroom, contact with other peers and the learner’s family outside the school environment) (Bronfenbrenner & Ceci 1994). In the exosystem, certain environments have an indirect influence on the learner, for example the education system, health services, media, work or a community organisation (Bronfenbrenner & Morris 2007). The inclusive education vision and mission statements and policies of a government, together with the underlying ethos to support learners who experience barriers to learning and development, form part of the macrosystem (Bronfenbrenner & Morris 2007; Meltzer & Muir 2022). The chronosystem relates to the concept of time of the child’s development. As children get older, they react differently to the environmental changes and may be able to determine how that change will influence them negatively or positively (Bronfenbrenner & Morris 2007).

The CHAT, which was originally developed by Vygotsky (1978) and later refined by Cole and Engeström (2007), involves the relationship between the human mind (what people think and feel) and activity (what people do) in understanding how they learn and develop (Emihovich & Lima 1995; Fletcher 2021; Sannino & Engeström 2018). The CHAT recognises the importance of the social and cultural environment and history in shaping experiences, emphasising activities that people engage in and interactions with others, such as teachers, peers and family members (Sannino & Engeström 2018). In the context of LSEN in school, Andrews et al. (2021) argue that the object (child) is shaped and directed by activity and that the child’s understanding of the activity within its context (e.g., understanding the value of physical activities in the PE class in a mainstream school) shapes the child’s learning and development. The historical and cultural part of the theory entail the understanding of the motives and the needs of the child within the activity system (Andrews et al. 2021). In the context of APE, before physical activity in a system (like the mainstream PE class) can take place, the hidden factors of the child’s disability over time should be understood by the teacher, which is where teacher’s experience in APE plays an important role (Andrews et al. 2021). Another aspect emphasised by the CHAT is the effect of tools or artifacts used by the person in the activity (Sannino & Engeström 2018). Tools such as language, teaching strategies, teacher knowledge of adapted physical activities and technology would influence the activity, development and learning of LSEN in APE. Through the lens of the CHAT, the ways people learn and grow can thus be understood by looking at the complex interactions between LSEN and APE teachers, their social and cultural environments and the tools they use (Hancock & Miller 2018; Sannino & Engeström 2018).

As one of the aims of the BRICS organisation is to improve the quality of education in the member countries, including special education to learners with disabilities (Van Jaarsveld 2021), the development and learning of LSEN within APE in each of the BRICS countries would be influenced by cultural and historical aspects of the ecological systems in each of the countries. Against this background and in view of the findings of various studies showing challenges in the implementation of APE in individual BRICS member states, the purpose of this study was to investigate the current state and status of APE in the BRICS countries, with the aim of making recommendations for best practice for the benefit of LSEN in these, and other, developing countries.

## Research methods and design

### Research design

This study used a qualitative approach in which document analysis was employed. The researcher analysed and interpreted the data within an interpretivist perspective in light of Bronfenbrenner’s theoretical framework, taking into account the different systems with regard to the learner, teacher, school, state, provincial and national policy makers, with the purpose of making recommendations for best practices in APE in the BRICS countries.

### Data collection

Data were gathered through the document analysis method, following the approach outlined by Harvey (2022). Various documents related to school curricula, education policies from government and organisational bodies, laws pertaining to education and training materials from higher educational institutions in each of the BRICS countries were obtained from the research databases EBSCOhost, Google Scholar and Web of Science. In addition, information was sourced from the educational websites of each BRICS country, as well as official government websites. The search terms ‘Adapted Physical Education’, ‘Adaptive Physical Education’ and ‘Inclusive Physical Education’ were employed across all documents. Relevant data concerning the condition and status of APE in primary and secondary schools in each specific country were identified, marked and coded. Documents that were available in English were used for the document analysis, while documents that were not in English were translated to English via ‘Google Translate’. Initially, 55 documents were retrieved, with 23 documents ultimately being utilised for the primary data analysis. These 23 documents and the key points analysed are shown in Table 1.

**TABLE 1:** Main analysed documents and key points.

Country	Document title	Type of document	Author(s)	Key points
Brazil	*Brazilian Law on the Inclusion of Persons with Disabilities* Guide for Physical Activity for the Brazilian population National Curriculum Guidelines for Undergraduate Courses in Physical Education	Public law National Governmental Guidelines National guidelines	Brazilian Presidency of the Republic (BPR 2015) Brazilian Ministry of Health (BMH 2021) Brazilian Ministry of Education (BME 2004)	Access to school games and sport Physical activity (PA) guidelines for learners with disabilities Teachers develop pedagogy, assessment Requirements for initial and continuing teacher training
Russia	*Physical Culture and Sports in the Russian Federation Federal Law No.329* Physical Culture and Sports Complex ‘Ready for Labor and Defence’ (GTO) Federal State Educational Standards	Federal law National Health & Fitness Program National Educational standards for teacher accreditation	The Russian Federation Council (RFC 2007) Ministry of Sport of the Russian Federation (MSRF 2017, 2023) National Centre for Public Accreditation (NCPA 2015)	Adaptive physical culture and sport by schools for people with disabilities Professional development of APE teachers Fitness guidelines, tests, norms for and assessment of disabled people Undergraduate APE teacher training
India	National Curriculum Framework for School Education Mainstreaming Health and Physical Education: Indian Manual for School Management Committees on Inclusion in Education *National Education Policy* PRASHAST	National school curriculum document National curriculum document National policy for school management committees National Education Policy Disability Screening Checklist	National Council of Educational Research and Training (NCERT 2023) Central Board of Secondary Education (CBSE 2022) National Council of Educational Research and Training (NCERT 2020) Ministry of Human Resource Development (MHRD 2020) Indian Ministry of Education (ME 2022)	Schools to adapt PA and sport for disabilities Adaptation guidelines for the PE strands Teachers develop assessment strategies Requirements for APE teacher undergraduate, professional development PE guidelines for teaching LSEN Assessment guidelines
China	*Sports Law of China* Curriculum Standards for Physical Education and Health in Compulsory Education (2022 ed) *Law of the People’s Republic of China on Protection of Disabled Persons* Decree on Regulations on Education for Individuals with Disabilities 14th Five-Year Plan Action Plan for Development and Improvement of Special Education *Compulsory Education Law of China.*	National Law National Curriculum Standards National Law National decree Public planning document Compulsory Education Law	China Standing Committee of the National People’s Congress (CSCNPC 2022) Ministry of Education (MOEPRC 2022) National People’s Congress (NPCPRC 2008) Ministry of Education (MOEPRC2021a) Ministry of Education (MOEPRC 2021b) Ministry of Education (MOEPRC 2006)	Schools to adapt activities for disabled learners to obtain compulsory education Adapting curriculum according to learners’ individual difference and abilities Professional teacher training requirements in special and inclusive education Assessment for school admission and transfer, and curriculum guidance Flexibility in assessment of compulsory education
South Africa	*Curriculum and Assessment Policy Statement* White Paper 6 on Special Needs Education Draft Learning Programme for Children with Severe to Profound Intellectual Disability *Draft Curriculum and Assessment Policy Statement for Learners with Severe Intellectual Disability for Life Skills PE* *Policy on Screening, Identification, Assessment, and Support (SIAS)* National Protocol for Assessment Gr. R-12 NWU 2024 Yearbook Faculty of Education (BEd)	National school curriculum National Policy Draft National Curriculum Program Draft National Curriculum Statement National Policy National Education Policy University Academic Yearbook	Department of Basic Education (SADBE 2011) Department of Education (SADE 2001) Department of Basic Education (DBE 2016) Department of Basic Education (DBE 2018) Department of Basic Education (DBE 2014) Department of Education (DBE 2012) North-West University, South Africa (NWU 2024)	Inclusivity principles in all subjects, also PE Adaptation for learners with disabilities Teacher training offered by the Ministry APE taught by qualified teachers Assessment for school admission and transfer, and curriculum guidance Curriculum and assessment adjustments managed at school level Requirements for grades not compromised APE modules in BEd degree programmes

Note: Please see the full reference list of the article, Engelbrecht, C., Du Toit, D., Van der Merwe, N. & Kemp, C., 2025, ‘Bridging policy and practice: Adapted physical education for special needs learners in BRICS’, *African Journal of Disability* 14(0), a1626. https://doi.org/10.4102/ajod.v14i0.1626">https://doi.org/10.4102/ajod.v14i0.1626, for more information.

### Data analysis

An inductive content analysis of the data extracted from the documents was carried out, following the qualitative content analysis guidelines proposed by Harvey (2022) and Maree et al. (eds. 2016). This process involved a thorough and systematic review of the document content, coding of the information and the identification of patterns and themes related to the state and status of APE in the educational documents of the BRICS countries (eds. Maree et al. 2016).

### Trustworthiness and credibility

Ensuring trustworthiness is crucial in qualitative research to validate the credibility of study findings. In this study, a triangulation approach was employed by comparing content across various policy or education documents within a country. To bolster the credibility of the findings, peer debriefing was undertaken. Apart from the initial analysis conducted by the primary author, an independent qualitative research expert was independently engaged in the document analysis process, and the outcomes were discussed in separate peer debriefing sessions. Moreover, to enhance the transferability of the study, an audit trail was meticulously conducted from the beginning of the analysis. Providing a comprehensive description of the research process further contributed to ensuring the study’s transferability (Harvey 2022; eds. Maree at al. 2016).

### Ethical considerations

This study was approved by the Research Ethics Committee of the Faculty of Education of the North-West University (reference no: NWU–01007–21–A2). Given that the study was based on the analysis of documents that were publicly available, there were no major ethical considerations, and the study was identified and accepted by the committee as an ethically ‘no-risk’ study.

## Results and discussion

The aim of this study was to investigate the state and status of APE in the BRICS countries. The themes that emerged from the document analysis were: *presence of the APE concept and guidelines, training and qualifications of teachers,* and *assessment*. For easier reference, the main findings and a discussion with regard to each country within that theme will consequently be presented.

### Presence of the adapted physical education concept and guidelines

*Article 28 in Chapter IV of the Brazilian Law* on the Inclusion of Persons with Disabilities states that it is mandatory for the public authority to ensure, create, develop, implement, encourage, monitor and evaluate ‘access of the disabled, on an equal basis, to games and recreational, sports and leisure activities in the school system’ (Brazilian Presidency of the Republic [BPR] 2015:Article 28).

In the national Guide for Physical Activity for the Brazilian population, some guidelines are provided for PE teachers in schools working with learners with disabilities:

It is important to ensure that all ‘students have access and participate in more and better physical education classes.’ (Brazilian Ministry of Health [BMH] 2021:46)

Therefore, teachers are urged to:

‘Encourage students with disabilities to actively participate in physical education classes.’ (p. 47)

‘Encourage, stimulate and let people with disabilities explore the environment as they wish at school.’ (BMH 2021:47)

Although inclusive education for LSEN in Brazil, based on public policies, was prioritised between the 2008 and 2018 and mandated the training of specialised teachers who have to conduct specialised services to LSEN in schools, Bezerra (2020) points out that there is a serious lack of such training opportunities for teachers in Brazilian universities, and that more policies should be developed to promote APE to learners with disabilities. Other studies (Hodge et al. 2018; Parente & Pessoa 2021; Santos & Mendes 2021) show that the inclusion process in Brazilian education is still being transformed, with the focus moving more to include LSEN in mainstream schools. Implementing inclusive education in schools, however, is still a challenging aspect in Brazil, and inclusion is still a controversial practice within the school system (Santos & Mendes 2021). In confirmation of the last statement, a recent decree issued by the Ministry of Education (10.502/2020 establishing a National Policy of Special Education [Shinohara 2021]) contradicts the principle of inclusive education by establishing a separate system of special schools, which allows Brazilian authorities to direct children with disabilities to special schools if they are considered to not developmentally benefit in regular inclusive schools and need multiple support services.

In Russia, PE (also called ‘Physical Culture’) is regulated by the *Physical Culture and Sports in the Russian Federation Federal Law No.329* (Mamazhanov 2022; Russian Federation Council [RFC] 2007). Chapter 3, Article 28 of this law reads that educational institutions are mandated towards:

Implementing measures for the development of physical culture and sport of the disabled, and persons with disabilities, adaptive physical culture, and adaptive sport in constituent territories of the Russian Federation. (RFC 2007, Chapter 3, Article 28)

Physical rehabilitation and social adaptation of disabled persons and persons with special needs by methods of adaptive physical culture and adaptive sport is carried out at rehabilitation centres, physical culture and sport centres for disabled persons, educational institutions and organizations for physical culture and sport. (RFC 2007, Chapter 3, Article 28)

The law further relates to the goal of adaptive physical culture and sport:

The development of physical culture and sports of persons with disabilities is aimed at increasing their motor activity and is an indispensable and determinable condition for the full rehabilitation and social adaptation of persons with disabilities. (RFC 2007, Chapter 3, Article 28)

Furthermore, the Physical Culture and Sports Complex ‘Ready for Labor and Defense’ [*Gotov k trudu i oborone* {GTO}] (MSRF 2017, MSRF 2023), a national health and fitness programme implemented by the Russian Ministry that forms the regulatory foundation of the country’s PE policy, also contains an adapted section with guidelines, tests and norms for learners with disabilities.

Russia’s policies thus aim to include all learners with disabilities in mainstream schools (RFC 2007; Valeeva 2015). Unfortunately, many LSEN are left out of the education system or accommodated in special schools because of economic reasons (Mamonova 2020; Valeeva 2015). Also, according to Van Jaarsveld (2021), inclusive policies and practices in different regions of Russia vary, and the accessibility of schools is often an obstacle. Other reasons for not introducing LSEN into mainstream schools include the insufficient training in APE of teachers in mainstream schools, and the availability of special needs experts, psychologists and social workers in special schools (Mamazhanov 2022; Valeeva 2015). Although inclusive education is part of curriculum reforms in Russia (MSRF 2023), researchers are of the opinion that APE should be developed further (Aksenov et al. 2023; Mamazhanov 2022).

In India, the National Curriculum Framework for School Education (National Council of Educational Research and Training [NCERT] 2023) states that schools must ensure equal opportunities for all learners to participate in PE and lists one of the requirements that:

Schools must ensure the participation of students with disabilities in Physical Education to the extent that is possible for them. This requires adapting play conditions through thoughtful accommodation or modification to enable them to participate. (NCERT 2023:421)

Some examples of such adaptations listed are: increasing the time to finish a run or allowing for individual differences in the skill levels expected of students with disabilities and modifications to game rules to ensure cooperative play among learners of different abilities (NCERT 2023). One of the learning standards of PE is even that ‘Students will also learn to modify a game or create new ones to include those who may have different needs and abilities’ (NCERT 2023:417). The participation of students in all activities stays, however, the responsibility of the teacher in order that ‘Games and activities must be chosen so that students of all genders and abilities can participate’ (NCERT 2023:438).

In the Mainstreaming Health and Physical Education (MHPE) curriculum for the secondary and senior secondary school levels in India, one of the main objectives of PE is stated as ‘To address the physical, psycho-social needs of CWSN (Children with Special Needs) in an integrated fashion’ (Central Board of Secondary Education [CBSE] 2022:5). In the MHPE, guidelines are also provided with regard to the inclusion of LSEN in the four strands of health and PE (sport and games, health and fitness, Social Empowerment through Work Education and Action [SEWA] and the health and activity card), with the common recommendation in every strand that ‘Students are free to innovate their own mechanisms for inclusion under the guidance of their class teachers’ (CBSE 2022:10).

Although inclusive PE is thus mandated by various laws and policies in India, studies report that these laws and prescriptions are often not implemented. Chennapragada (2021) points out that one of the causes of this is that the different types of schools in India are not all affiliated under the two main educational boards (the CBSE and the NCERT), leading to a lack of national standards for PE and APE. In the study done by Chennapragada (2021) involving 18 learners with physical disabilities from Telugu-speaking states, the participants reported that they were generally not able to access PE in their schools or sport activities in their communities, and that their PE teachers showed a lack of knowledge and training to work with LSEN. Similar findings were reported by parents of learners with cerebral palsy (CP) in the study done of Paleeri (2020).

In the *Sports Law of China* (China Standing Committee of the National People’s Congress [CSCNPC] 2022), Article 18 states that:

Schools must offer Physical Education and make it a subject for assessing students’ academic performance. Schools shall create conditions for organizing sports activities suitable to the special features of students who are in poor health or disabled. (CSCNPC 2022:Article18)

In the recently released Curriculum Standards for Physical Education and Health in Compulsory Education (2022 edition) (Ministry of Education of the People’s Republic of China [MOEPRC] 2022:6), which forms part of major reforms with regard to PE and health education in Chinese schools, one of the six aspects that are emphasised is: ‘paying attention to the individual differences of students’. The standards also include in-depth details on how PE teachers should attend to differences between individual school learners in the content they teach, as well as their teaching strategies, for example:

… the selection and design of teaching content should fully take into account the developmental characteristics, physical condition, sports foundation, interests and needs of students … to ensure the basic, diversity and systematic nature of teaching and guiding students to be physically active. (MOEPRC 2022:121)

These guidelines correlate with the requirements of adapting for learners with disabilities so that they can experience the same quality of education, as set out in the *Law of the People’s Republic of China on Protection of Disabled Persons* (National People’s Congress of the People’s Republic of China [NPCPRC] 2008). Also, in the revised versions of the Compulsory Education Curriculum Standards for deaf schools, blind schools and schools for intellectually challenged learners, which were released by the (MOEPRC) in 2016, some guidelines for ‘PE and Health’ have been included in the curriculum standards for children with disabilities (Liang et al. 2022).

Although the Chinese government has placed renewed focus on the educational rights of people with disabilities as well as on the PE curriculum, several studies have shown that inclusive PE has developed slowly and several challenges to the implementation of APE exist (Liang et al. 2022; Xu, Cooper & Sin 2018). These challenges include, among others: inadequate funds, mainstream teachers’ knowledge and training in inclusive education, inadequate curriculum modification, peers’ unfavourable attitudes towards LSEN and an ineffective evaluation system for LSEN in the regular classroom (Liang et al. 2022; Xu et al. 2018). According to Wang et al. (2020), China does not have enough APE teachers to provide PE to LSEN and more APE teachers should be trained to improve the quality of APE for LSEN. Xue et al. (2023) point out that, although the increased focus on the rights of disabled learners has led to improved living standards of disabled Chinese learners, discrimination and social stigma against disability remain in Chinese societies.

In South Africa, ‘Life Orientation’ (called ‘Life Skills’ in the lower grades) is a compulsory subject in the national school curriculum, consisting of six topics (Development of the self in society, Social and environmental responsibility, Democracy and human rights, Careers and career choices, Study skills and Physical Education) (South African Department of Basic Education [SADBE] 2011). Inclusion in PE or APE is not stipulated in the PE curriculum in the national curriculum policy, named the Curriculum and Assessment Policy Statement (CAPS), but inclusivity is required to be applied in all six the topics of Life Orientation, as stated in the CAPS:

Inclusivity should become a central part of the organisation, planning and teaching at each school. This can only happen if all teachers have a sound understanding of how to recognise and address barriers to learning, and how to plan for diversity. (SADBE 2011:5)

In the CAPS (SADBE 2011), it is further recommended that teachers and schools apply the principles of inclusion as stipulated in the Guidelines for Inclusive Teaching and Learning (SADBE 2010) as well as in the White Paper 6 (SADE 2001) in every topic of Life Orientation. Although no specific guidelines pertaining to PE are provided in the latter two documents, very specific PE guidelines are provided (also for learners with other disabilities) in the Draft Learning Programme for Children with Severe to Profound Intellectual Disability (SADBE 2016) and the draft Curriculum and Assessment Policy Statement for Learners with Severe Intellectual Disability for Life Skills Physical Education (CAPS LSIDPE) (SADBE 2018). In the CAPS LSIDPE, it is stated that these guidelines can be used for teaching PE to other learners with disabilities as well, and that:

A learner with a severe intellectual disability must be supported to optimally participate in physical education, as their physical wellbeing and ability to move supports their ability to learn and develop to his/her maximal potential. (SADBE 2018:7)

Limited research exists on PE and participation of LSEN in South Africa. Bantjies et al. (2015) conducted a study involving 15 learners with CP between the ages of 12 and 18 years in the Western Cape province and their participation in physical activity, including PE. The researchers concluded that the participating LSEN felt isolated as they were not included in team sports, because coaches and teachers were unable to accommodate their particular level of impairment (Bantjies et al. 2015). Bantjies et al. (2015) also pointed out that LSEN’ participation was limited by the availability of resources and facilities, and that some adapted physical activities were only offered to LSEN from a certain age onwards, which placed a restriction on their participation and exposure to PE and sport. According to the findings of Burnett (2021) in a national study on the state and status of PE, LSEN-schools (special schools) in South Africa follow a highly valued rehabilitative approach where the focus is on health-inducing physical activities presented by specialised staff. Challenges reported for LSEN-schools in South Africa in the study, include inadequate curriculum content, a lack of clear guidelines for implementation, a lack of adequately trained educators to deliver adapted activities, and a lack of opportunities for LSEN to participate with able-bodied learners (mainstreaming) (Burnett 2021). Another obstacle for the proper implementation of inclusive education is the economic imbalance and social exclusion that still exist in the country (Burnett 2021). Although the LSEN-schools in South Africa generally have small classes and PE is provided by trained specialists to ensure that the individual needs of the learners are met, at some special schools, and various mainstream schools, LSEN do not participate in PE at all because of insufficient structures and trained PE teachers (Oladunni, Lyoka & Goan 2015).

### Training and qualifications of teachers

In Chapter IV, *Article 27 of the Brazilian Law* on the Inclusion of Persons with Disabilities, it is stated that schools and higher education institutes are responsible for ‘… adoption of inclusive pedagogical practices from the programs of initial and continuing teacher training in order to offer continuous training for specialized educational services’ (BPR 2015, Chapter IV, Article 27) and ‘… training and availability of teachers for specialized educational services, translators and interpreters of Libras, interpreters and support professionals’ (Chapter IV).

According to the National Curriculum Guidelines for Undergraduate Courses in Physical Education, PE teachers should be trained:

To diagnose the interests, expectations and needs of people (children, young people, adults, the elderly, people with disabilities, special groups and communities) in order to plan, prescribe, teach, guide, advise, supervise, control and evaluate projects and programs of physical, recreational and sports activities from the perspective of prevention, promotion, protection and rehabilitation of health, of the cultural training, motor education and re-education, physical-sports performance, leisure and other fields that provide or will provide opportunities for the practice of physical, recreational and sports activities. (BME 2004:2)

In their overview study of APE teacher training, Gonçalves et al. (2020) conclude that although progress has been made in improving the APE training of PE pre-service teachers in various tertiary institutes in Brazil, shortages with regard to the specificity and volume of the training still persisted. The study done by Hodge et al. (2018) among Brazilian APE teachers also showed that the main challenge to inclusion in PE in mainstream schools was the professional and pre-graduate training of the teachers. Even though the teachers in the study by Hodge et al. (2018) believed in inclusive APE, they struggled to implement the requirements of the laws for inclusive education in the PE class because of a lack of training, knowledge and consequently, a lack of confidence in working with LSEN. These findings are supported by those of Barros et al. (2023), in a systematic review of APE in Brazilian public schools, where the findings showed that there was still a lack of specialised teacher training in APE in the country.

Teacher training in APE is available at various Russian universities in the form of a Bachelor’s, Masters’ or special degree, as regulated by the Federal State Educational Standards (Baranov et al. 2018; National Centre for Public Accreditation [NCPA] 2015). With regard to the continuing professional development of teachers, Chapter 3, Article 30 of the *Federal Law on Physical Culture and Sports* in the Russian Federation decrees that:

The state creates the conditions for professional development at least once every five years for employees of the executive branch in the field of physical culture and sport, employees of physical culture and sports organizations, as well as employees of state employees. (RFC 2007, Chapter 3, Article 30)

Baranov et al. (2018) studied Federal Laws related to inclusive education, including governmental teacher training necessary to implement the federal laws for learners with disabilities in educational institutes. Although Baranov et al. (2018) found that laws and policies were in place to provide sufficiently trained teachers for LSEN in mainstream Russian schools, all schools do not necessarily have teachers who are trained with regard to the specific needs of the learner. Concerning professional development of special needs teachers. Hanssen, Hansen and Strom (2021) found that although the Ministry of Education has integrated inclusive education modules into teacher education, a strategic approach to and clear requirements regarding the professional development of special needs teachers are still lacking. Hanssen and Erina (2022), in a study involving 60 parents of LSEN from across Russia, also concluded that special needs teachers are often not adequately trained to teach LSEN.

In the Indian Manual for School Management Committees on Inclusion in Education (NCERT 2020:36), it is recommended that there is an additional ‘part time instructor for art education, health and physical education, and work education’ in classes of 35 learners or more. Guidelines for teaching learners with disabilities, including physical therapy and physical movement, are also provided (NCERT 2020).

In the National Education Policy (NEP) of India, it is also stated that ‘the awareness and knowledge of how to teach children with specific disabilities (including learning disabilities) will be an integral part of all teacher education programmes …’ (MHRD 2020:27) and:

Each teacher will be expected to participate in at least 50 hours of CPD opportunities every year for their own professional development, driven by their own interests. CPD opportunities will, in particular, systematically cover the latest pedagogies regarding foundational literacy and numeracy, formative and adaptive assessment of learning outcomes. (p. 22)

The NEP further stipulates that the 4-year BEd degree, which will include ‘training in time-tested as well as the most recent techniques in pedagogy, including pedagogy with respect to foundational literacy and numeracy, multi-level teaching and evaluation, teaching children with disabilities’ (MHRD 2020:23), will be the minimum qualification for a teacher in 2023, although short courses and certificates for teaching learners with disabilities can also be offered by schools and higher education institutes.

Although the Manual for School Management Committees on Inclusion in Education (NCERT 2020), the NEP (MHRD 2020) and the National Curriculum Framework for School Education (NCERT 2023) provide guidelines and requirements for educators who teach LSEN in India, as stated precedingly, Gale et al. (2022) found that the availability of qualified and trained PE teachers is stated as a challenge to the quality implementation of the PE curriculum. Researchers recommend that the Ministry of Education should use respectful, capacity-building approaches to teacher training, rather than ‘communication of bold requirements in policy statements’ (Gale et al. 2022:35). Another suggestion, provided by the NEP (MHRD 2020) concerning the lack of trained teachers, is the sharing of teachers of specialised subjects such as APE, across schools in a state government.

In China, various policies and governmental documents provide guidelines and prescriptions for LSEN teacher training. According to Chapter II, Article 22 of the Decree on Regulations on Education for Individuals with Disabilities:

Regular schools that enrol students with disabilities shall arrange for teachers exclusively engaged in the education for individuals with disabilities … so as to ensure that the students with disabilities can equally participate in educational and teaching activities as well as various activities organized by the schools. (MOEPRC 2021a, Chapter II, Article 22)

Beside the schools, the responsibility of training special needs’ teachers also lies with county governments, as stated in Chapter VI, Article 40 of the Decree:

People’s governments at or above the county level shall place emphasis on educating and training teachers specializing in education for individuals with disabilities and adopt measures to gradually raise their status and benefits, improve their working environment and conditions, and encourage them to engage in education for individuals with disabilities. (MOEPRC 2021a, Chapter VI, Article 40)

The implementation of compulsory continuing training of principals and teachers of special and ordinary schools and subsidised allowances for teachers engaging in special education are also mandated in the 14th Five-Year Plan Action Plan for Development and Improvement of Special Education (MOEPRC 2021b) and in Article 31 of the *Compulsory Education Law of the People’s Republic of China* (CSCNPC 2006).

Chinese policies and governmental decrees thus show strong support for the training and uplifting of special education teachers. In a systematic review of APE in China, Liang et al. (2022) list nine universities in China that offer high-level APE teacher training programmes, of which two offer postgraduate programmes specialising in APE. However, in the study done by Liang et al. (2022), the lack of knowledge and training and attitudes of APE teachers towards teaching PE to LSEN were identified as two major factors negatively influencing the inclusion of LSEN in APE classes in ordinary as well as special schools. Similarly, Xue et al. (2023), in a study involving 286 APE teachers, found low levels of inclusive education competency of the participating APE teachers, and Li et al. (2022), in a study done in Singapore, recommended the further training of PE teachers to enhance the attitudes of peers without disabilities towards LSEN.

In South Africa, the White Paper 6 decrees that:

Classroom educators will be our primary resource for achieving our goal of an inclusive education and training system. This means that educators will need to improve their skills and knowledge, and develop new ones. (SADE 2001:18)

[*I*]n collaboration with our provincial departments of education, the Ministry will, through the district support teams, provide access for educators to appropriate pre-service and in-service education and training and professional support services. (SADE 2001:29)

In the South African Policy on Screening, Identification, Assessment and Support (SIAS) document (SADBE 2014:22), the training of teachers is also mandated, with the quantity and intensity of training sessions increasing from low and medium to high levels of support provided by the Department of Basic Education. In the CAPS for learners with intellectual disabilities (SADBE 2018), it is also prescribed that ‘An appropriately qualified teacher … is required to teach Life Skills Physical Education’ (SADBE 2018:7).

While inclusive education is mandated in pre-service teacher education by the legislative framework for teacher education in South Africa and most universities offer inclusive education modules or sections of modules in their BEd degrees (Rusznyak & Walton 2019), only a few universities provide pre-service teacher training in APE (NWU 2024; UP 2024), and no evidence of official in-service teacher training in APE could be found. In alignment with this, the lack of pre-service as well as in-service teacher training has been identified as a barrier to the implementation of quality PE to LSEN in South Africa in the studies done by Burnett (2021) and Bantjies et al. (2015).

### Assessment

Although not providing details for assessment in APE, the Physical Activity Guide for the Brazilian Population urges PE teachers to ‘Seek to improve the quality of their classes through training and exchange of experiences on the curriculum, pedagogical practices and assessment’ (BMH 2021:3).

Costin and Pontual (2020) point out that even though the Brazilian Law on the Inclusion of Persons with Disabilities (BPR 2015) prescribes that LSEN should be included in PE assessment according to PE-specific competencies and skills set out in the national school curriculum, the mostly decentralised education system of Brazil where regional governments must develop their own curricula and learner assessments creates a challenge for assessment in PE for learners with and without disabilities.

The tests and norms provided in the Russian Adapted GTO complex for people with disabilities (Aksenov et al. 2023; MSRF 2023) are used extensively for the assessment of the physical and motor fitness of LSEN in PE. The complex comprising of compulsory and optional tests and norms available for people with disabilities is adapted for different age groups. Moreover, it has been tested for learners with different disabilities such as visual and auditive impairments and intellectual and physical disabilities (Aksenov et al. 2023; Rubtcova & Pavenkov 2018). Although some researchers recommend further refinement of the GTO complex for people with disabilities (Rubtcova & Pavenkov 2018), other studies (Aksenov et al. 2023; Mamazhanov 2022) show positive results and feedback with regard to the use of the system in APE.

In India, the PRASHAST (Pre Assessment Holistic Screening Tool), a Disability Screening Checklist for Schools published by the Indian Ministry of Education (ME 2022), can be used by regular and specialist teachers to set a baseline for sequential assessments to monitor learners’ progress in PE. Furthermore, the NCERT includes ‘… designing a range of activities and sports for all students, including those with disabilities’ (NCERT 2023:438), as part of the pedagogy and assessment that PE teachers have to apply in their teaching. With regard to the four strands of PE in the MHPE curriculum, LSEN are ‘free to innovate their own mechanisms for inclusion under the guidance of their class teachers’ (CBSE 2020:10). Although LSEN are required to submit a portfolio of evidence that they have participated in the subject Health and Physical Education to be able to write the board exams at the end of Grade XII (CBSE 2020; NCERT 2023), no specific guidelines are provided for the assessment of PE among LSEN.

The above-mentioned requirement of the NCERT (2023) that PE teachers must adapt activities and the assessment thereof for LSEN themselves, together with the absence of uniform national standards in PE in India, are considered to be some of the obstacles to the implementation of quality APE in India (Chennapragada 2021).

In China, Chapter II, Article 20 of the Decree on Regulations on Education for Individuals with Disabilities, mandates that an expert committee consisting of the administrative departments of education, public health, civil affairs and disabled persons’ federations in the counties, should:

[*A*]ssess the physical conditions of school-age children and adolescents with disabilities as well as their abilities to receive education and adapt to school life, so as to give advice on their school admissions or transfers; they may also provide consulting services and give advice on compulsory education for individuals with disabilities. (MOEPRC 2021a, Chapter II, Article 20)

As Chinese learners have to complete a PE exam as part of their final Grade 12 exam (the ‘Gaokao’), LSEN also have to complete the PE exam, but with adaptations as mandated by governments at the county level:

For the students with disabilities who receive compulsory education in regular schools by means of learning in regular classrooms, the curriculum designs, curriculum standards, and teaching materials for regular compulsory education may be applied, but appropriate flexibility may be allowed in terms of academic requirements. (MOEPRC 2021b, Chapter II, Article 23)

In special schools, the curriculum designs and curriculum standards (including assessment guidelines) for special education are developed by the administrative department of education under the State Council (MEPRC 2021, Article 25), and flexibility in the application of these is also mandated in *Article 23 of the Law on the Protection of Persons with Disabilities*:

[*A*]llowing appropriate flexibility in determining the curricula, teaching materials and methods and the age requirement for admission and graduation for special education. (NPCPRC 2008:Article 23)

Although guidelines for the teaching of *PE and Health* to LSEN are provided in the Chinese school curriculum documents for deaf, blind and intellectually challenged learners, no specific guidelines for the assessment and grading of LSEN in APE could be found in Chinese policies, which have been identified by APE teachers as one of the factors that negatively influences inclusive PE in China (Liang et al. 2022).

In South Africa, the SIAS document addresses the assessment of LSEN by referring to:

Curriculum and assessment adjustments required to allow learners at multiple levels of functioning to access the curriculum and assessment tasks best suited to their needs. Such accommodations can be managed at school or classroom level. (SADBE 2014:20)

However, with regard to the assessment of LSEN in the National Protocol for Assessment Grades R-12, the SADBE emphasises that:

The minimum requirements for achieving grades, as spelt out in the National Curriculum Statement (Grades R – 12), may not be compromised. However, within a flexible learner-based and learner-paced approach to the curriculum, all learners could be enabled to achieve their full potential irrespective of whether or not the end result will be a final certificate. (SADBE 2012:31)

The practical assessment of PE among LSEN is also prescribed in the draft curriculum for learners with intellectual disabilities (CAPS LSIDPE) (SADBE 2018), where it entails a twice-quarterly assessment of the learner’s participation in PE as well as his or her performance of movements, at the level at which they are capable of performing. The CAPS LSIDPE provides specific guidelines on the assessment of the gross-motor and perceptual-motor developmental levels of learners with intellectual disabilities, and declares that these guidelines can be used for LSEN with other disabilities as well (SADBE 2018). However, the CAPS LSIDPE was only a draft document and was never finalised after comments from stakeholders had been received (SADBE 2018). Assessment in PE was one of the challenges, along with inadequate PE content and implementation guidelines, reported by various teachers teaching APE in the national study done by Burnett (2018).

### Recommendations

In view of the importance and value of physical activities and sport within APE for the holistic development and rehabilitation of LSEN, the development of more specific guidelines and standards for adaptations and assessment in the content areas of the PE curriculum, as well as specific APE pre-service and in-service teacher training, is strongly recommended with regard to the national policies and curricula of the BRICS nations. Furthermore, more comprehensive and robust efforts from the education ministries to implement the policies and laws with regard to LSEN and teachers in APE are required.

### Strengths and limitations

The strengths and limitations of this study should be taken into account when considering the findings. To our knowledge, this is the first qualitative study to investigate the state and status of APE in the policies of BRICS countries. Strengths of this research further include the thoroughness of the document collection and analysis, offering a vast and diverse range of data and showcasing various viewpoints on APE in national policies. However, there are some limitations to consider. Some of the documents had to be translated from Russian or Portuguese into English, which may have resulted in subtle nuances being lost in translation. Moreover, as Harvey (2022) pointed out, documents can sometimes contain biases or selective information as they were created for specific reasons. To mitigate this, the researchers carefully assessed the context and purpose of each document (which were available in English).

## Conclusion

The purpose of this study was to investigate the state and status of APE in the BRICS countries by means of the analyses of government education policies and curricula.

The findings show that all BRICS nations have policies in place that mandate the inclusion of LSEN in PE and sport in mainstream schools and which recognise the importance of physical activities within PE and sport for more severely disabled learners in special schools. However, specific guidelines for adapting activities in PE could only be found in governmental documents of Russia, India and China. In Brazil, although general physical activity guidelines for people with disabilities are provided, the national school curriculum requires teachers to make adaptations for LSEN in PE and sport without being specific. Similarly, the Life Orientation curriculum (in which PE is embedded) in South Africa decrees the inclusion of LSEN in mainstream schools but does not provide specific guidelines for APE (although a draft curriculum for learners with severe intellectual disabilities has been published).

With regard to teacher training and qualifications, the education policies of all the BRICS countries require teachers of LSEN to be trained and qualified in special or inclusive education. Specialised undergraduate teacher training in APE can be received at universities across all the BRICS countries although it is limited to a few universities in South Africa. Moreover in India, other APE qualifications (short courses and certificates) are also available. In all the BRICS countries, the in-service development of teachers who teach LSEN is mandated, but specific requirements for APE professional development are mostly lacking. Furthermore, literature with regard to APE teacher training in all the BRICS countries indicates a lack of knowledge, confidence and training among many in-service APE-teachers, showing challenges in the implementation of teacher training policies.

The assessment of LSEN within APE is integrated in general subject guidelines for pedagogy of all BRICS members’ school curricula. In general, education policies and curricula require that LSEN follow the standard curriculum content with flexibility or adaptations in the assessment thereof, but specific requirements for assessment in APE are lacking. Specific fitness tests and norms for learners with disabilities in APE could only be found in the GTO-complex of Russia, while some assessment guidelines are included in the Chinese curricula for auditory, visually and intellectually challenged learners. In the South African curriculum, there is a draft learning programme for learners with intellectual disabilities. More specific guidelines and requirements for the assessment of LSEN in APE are thus needed, as confirmed by studies in all the BRICS member countries.

A summary of the findings showing similarities, differences and recommendations with regard to APE in BRICS countries is shown in Table 2.

**TABLE 2:** A general summary of the findings: Similarities, differences and recommendations regarding adapted physical education in BRICS.

Similarities	Differences	Recommendations
**Presence of the APE concept and guideline**		
Inclusive education policies are in place in all of the BRICS nations, where PE is included as part of normal school subjects. Brazil, Russia, China and South Africa have central education boards governing a national school curriculum.	Brazilian government supports the education of LSEN in special schools to a larger extent compared to other BRICS countries. Specific APE curricula exist in Russia and China, while in Brazil, India and South Africa general guidelines for inclusion in all subjects, also PE, occur in curricula. India has a range of different school education boards governing school curricula.	Inclusive education should be mandated equally in all BRICS countries. Standardised national APE curricula, with specific guidelines and content for APE in each country, should be developed and endorsed by all educational boards in the BRICS nations.
**Training and qualifications of teachers**		
In all BRICS countries except for South Africa, APE teacher training is part of pre-graduate teacher training programmes, postgraduate APE specialisation is available in tertiary institutions, and continuing professional training is mandated for special needs teachers, which can include APE. In all BRICS countries, a shortage of specialised APE teachers in mainstream schools is reported.	In South Africa, APE teacher training is generally not part of PE teacher training, postgraduate teacher training in inclusive education rarely includes PE, and continuing education is optional for teachers.	APE should form part of all pre-graduate teacher training programmes, and APE continuing education should be mandatory. Governments should increase their support of the training of teachers specialising in APE, by encouraging and offering more training opportunities (pre-graduate and postgraduate).
**Assessment**		
In Brazil, India, China and South Africa, policies only recommend general adaptations for LSEN regarding normal PE assessment according to the PE curricula.	In Russia, specific tests and norms are provided in the GTO for LSEN.	Within the standardised national APE curricula in BRICS countries, specific assessment guidelines, tests and norms should be provided for LSEN.

PE, physical education; APE, adapted physical education; BRICS, Brazil, India, China and South Africa.

In the context of Bronfenbrenner’s EST (Bronfenbrenner & Morris 2007) and the CHAT (Sannino & Engeström 2018), the exosystems, including the national policies and curricula of the BRICS nations as well as their implementation, form part of the cultural and historical contexts within which LSEN learn and develop and have an indirect influence on the physical and other aspects of development of LSEN in APE. As the implementation of the national policies and curricula is not optimal in all the countries, the mesosystems of the BRICS countries are also negatively affected as they include the training and functioning of APE teachers. The shortage of trained teachers, in turn, affect the tools of teaching strategies, experience and knowledge, which influence the activities and learning of LSEN in the APE class and ultimately the rehabilitation and development of LSEN in the microsystems.
